# Assessing Telehealth in Palliative Care: A Systematic Review of the Effectiveness and Challenges in Rural and Underserved Areas

**DOI:** 10.7759/cureus.68275

**Published:** 2024-08-31

**Authors:** Kamal Y Ghazal, Shreya Singh Beniwal, Avleen Dhingra

**Affiliations:** 1 Palliative Care, King Fahad Medical City, Riyadh, SAU; 2 Medicine and Surgery, Lady Hardinge Medical College, New Delhi, IND; 3 Anesthesia, Dayanand Medical College and Hospital, Ludhiana, IND

**Keywords:** virtual care, remote healthcare, rural healthcare, palliative care, telehealth

## Abstract

The integration of telehealth into palliative care has garnered significant attention due to its potential to enhance both access and quality of care, particularly for patients in rural and underserved areas. This interest stems from the need to address geographical and logistical barriers that traditionally hinder palliative care delivery. Despite its potential benefits, the effectiveness of telehealth and the challenges associated with its implementation remain underexplored, necessitating further investigation. This study aims to critically evaluate the effectiveness of telehealth in palliative care by focusing on several key areas: its impact on access to care, symptom management, patient satisfaction, and cost-effectiveness. To achieve this, a systematic review was conducted, synthesizing data from various studies that investigated telehealth interventions within palliative care settings. The review employed a comprehensive search strategy across electronic databases, concentrating on randomized controlled trials (RTCs) published between 2014 and 2024. To ensure the reliability of the findings, low-quality and unrelated studies were excluded, and the remaining studies were meticulously analyzed for bias and methodological quality. The review's findings indicate that telehealth significantly enhances access to palliative care, allowing patients to receive timely and appropriate care without the need for extensive travel. It also improves symptom management and patient satisfaction, aligning to provide patient-centered care. Additionally, telehealth is cost-effective by reducing expenses associated with travel and in-person visits. These benefits highlight telehealth's potential to address some of the critical challenges in palliative care delivery. Despite its advantages, implementing telehealth in palliative care is not without challenges. Technological barriers, such as inadequate infrastructure and device limitations, pose significant hurdles. Integration issues, including the need for seamless incorporation into existing care systems, and varying levels of digital literacy among patients and caregivers, also impact the effectiveness of telehealth. Addressing these challenges is crucial for optimizing telehealth's implementation. Ensuring that telehealth solutions are accessible, user-friendly, and well-integrated into care practices is essential for fully leveraging its potential benefits.

## Introduction and background

Telehealth refers to using technology to provide healthcare services remotely. It includes telemedicine, which involves real-time video consultations between patients and healthcare providers, enabling diagnosis, treatment planning, and follow-up without physical presence [1]. Remote monitoring uses electronic devices to monitor patients' health metrics, allowing healthcare providers to track their condition and adjust treatment plans [2]. Virtual consultations, conducted via video or phone calls, allow patients and healthcare providers to interact in real time, enabling routine check-ups, specialist consultations, and chronic condition management [3].

Palliative care is a specialized medical approach that aims to improve the quality of life for patients with serious illnesses [4]. It involves symptom management, holistic support, and improving the overall quality of life by addressing the patient's and family's needs [5]. This includes supporting decision-making, advanced care planning, and facilitating communication between patients, families, and the healthcare team [6]. Care coordination ensures that all aspects of a patient's care are well coordinated, ensuring a seamless and supportive experience for the patient [7].

Telehealth offers numerous benefits in palliative care, particularly in rural and underserved areas. It increases accessibility by providing remote consultations and follow-ups, reducing the need for long travel times [8]. This facilitates continuous care by enabling regular virtual check-ins and monitoring, ensuring consistent oversight of the patient's condition and prompt management of symptoms [9]. Telehealth also optimizes healthcare resources by allowing specialists to reach a broader patient base without physical relocation, distributing expertise and services across wider geographic areas [10].

Improved patient and family support is another benefit of telehealth [11]. Virtual consultations and support groups provide emotional and practical support, helping families manage the complexities of serious illness and navigate the healthcare system more effectively [12]. Telehealth can lead to cost savings by reducing travel, hospital admissions, and the overall burden on healthcare systems [13]. Flexibility and convenience are also advantages of telehealth. Patients can schedule appointments from their homes, which is particularly beneficial for those with mobility issues or severe illness [14].

Telehealth allows healthcare providers to expand their reach and deliver care to patients who might otherwise be underserved [15]. Timely interventions and continuous monitoring are crucial for managing complex symptoms and making prompt adjustments to care plans [16]. Telehealth platforms facilitate the collection and analysis of patient data, allowing for better tracking of health trends, outcomes, and intervention effectiveness [17]. These data can inform future care strategies and contribute to research in palliative care. Lastly, telehealth supports better care coordination by allowing multiple healthcare providers to collaborate remotely, leading to more integrated and holistic care [18]. Overall, telehealth offers numerous advantages for palliative care in rural and underserved areas, enhancing accessibility, continuity, and quality of care, optimizing resources, and reducing costs [19].

Telehealth in palliative care is crucial for addressing access gaps in rural and underserved areas. By bridging the gap between long travel times and limited local resources, telehealth can enhance the quality of life for patients with serious illnesses. This research will also inform resource allocation and policy-making, guiding the development of training programs for healthcare providers. By identifying specific challenges in rural and underserved areas, the study aims to suggest targeted solutions to make telehealth a more viable and patient-centered option. This systematic review aims to assess the effectiveness and challenges of telehealth in palliative care, specifically in rural and underserved areas.

This systematic review aims to assess the effectiveness and challenges of telehealth in palliative care, specifically in rural and underserved areas.

## Review

Methods

To structure our investigation, we employed the PICO (Population (P), Intervention (I), Comparison (C), and Outcome (O)) framework. Additionally, we examine implementation factors including integration into existing care models, cost-effectiveness, and training needs. This structured approach allowed us to explore not only the efficacy of telehealth in improving palliative care but also the obstacles encountered in these areas, to identify solutions to enhance telehealth services. The details of the PICO framework are summarized in Table 1. The PICO framework in Table 1 provides a structured approach to formulating research questions by outlining the Population (P), Intervention (I), Comparison (C), and Outcome (O) of interest.

PICO Framework

Table 1 explains the application of the PICO framework to the review. It details the specific components of the framework - population, intervention, comparison, and outcome - used to define the scope of the review and guide the selection criteria for studies. This framework ensures that the research question is clearly structured and that included studies are relevant to the defined parameters.

**Table 1:** PICO framework PICO: Population (P), Intervention (I), Comparison (C), and Outcome (O)

Component	Description
Population (P)	Patients receiving palliative care in rural and underserved areas
Intervention (I)	Telehealth services
Comparison (C)	Traditional in-person palliative care
Outcomes (O)	Effectiveness of telehealth in improving the outcome and identifying challenges to implementation

Eligibility Criteria for Selection of Studies

Table 2 explains the eligibility criteria for systematically ensuring that the included studies are relevant, high quality, and address the review's research question comprehensively.

**Table 2:** Eligibility criteria of studies

Domain	Inclusion criteria	Exclusion criteria
Population	Studies involving adults with palliative care needs, specifically in rural and underserved areas.	Studies that do not specifically focus on adults with palliative care needs in rural or underserved areas.
Intervention	Research on telehealth interventions for palliative care.	Research not related to telehealth interventions for palliative care.
Comparison	Studies comparing telehealth interventions to standard in-person palliative care or no telehealth intervention.	Studies without comparison to standard in-person care or no telehealth intervention.
Outcome	Valuations of the effectiveness of telehealth in improving the quality of care, addressing challenges, and identifying barriers to implementation.	Studies that do not evaluate the effectiveness of telehealth, or do not address challenges and barriers specific to rural or underserved areas.
Study Types	Includes randomized controlled trials (RCTs), meta-analyses, cohort studies, case-control studies, and observational studies with appropriate control groups.	Excludes non-peer-reviewed publications, editorials, commentaries, letters, conference abstracts, and animal studies.
Language	Studies published in English or with available translations.	Studies not available in English.
Publication Date	Studies published in peer-reviewed journals within the last 10 years to ensure relevance and currency.	Studies published before a specified cut-off date (e.g., 2014) to ensure relevance.

Search Strategy

The full search strategy used to identify relevant articles is detailed in Table 3. It includes Medical Subject Headings (MeSH) terms and text words related to the research process beginning with sourcing studies from three key databases: MEDLINE (PubMed), Scopus (Elsevier), and The Cochrane Library (Wiley), which collectively offer a broad and comprehensive range of biomedical literature.

**Table 3:** Search strategy

Database	Query	Records retrieved	Dates
MEDLINE via PubMed website (pubmed.ncbi.nlm.nih.gov)	("Telehealth" OR "Telemedicine" OR "Virtual Care" OR "Remote Monitoring") AND ("Palliative Care" OR "Palliative Medicine") AND ("Rural" OR "Underserved Areas" OR "Remote Areas") AND ("Effectiveness" OR "Outcomes") AND ("Challenges" OR "Barriers")	176	10-07-2024
Scopus Via Scopus website (scopus.com)	(TITLE-ABS-KEY("Telehealth" OR "Telemedicine" OR "Virtual Care" OR "Remote Monitoring") AND TITLE-ABS-KEY("Palliative Care" OR "Palliative Medicine") AND TITLE-ABS-KEY("Rural" OR "Underserved Areas" OR "Remote Areas") AND TITLE-ABS-KEY("Effectiveness" OR "Outcomes") AND TITLE-ABS-KEY("Challenges" OR "Barriers"))	272	12-07-2024
Cochrane Library via The Cochrane Library website (cochranelibrary.com)	("Telehealth" OR "Telemedicine" OR "Virtual Care" OR "Remote Monitoring") AND ("Palliative Care" OR "Palliative Medicine")	160	14-07-2024

Study Selection

**Figure 1:**
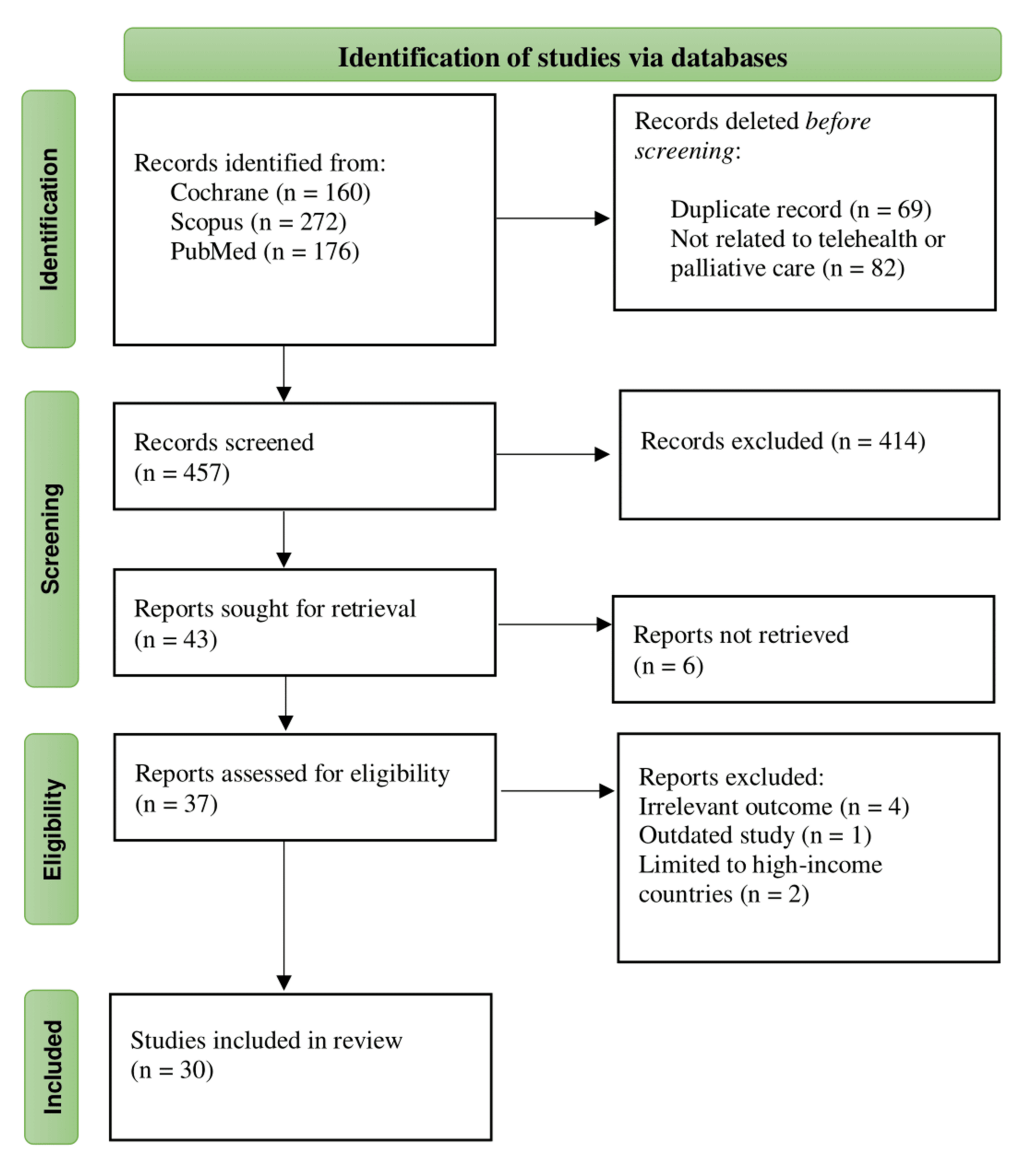
PRISMA flow chart PRISMA: Preferred Reporting Items for Systematic Reviews and Meta-Analyses

All retrieved references were imported into Endnote version 21 to organize the literature and remove any duplicate entries. For the study screening and selection process, Rayyan (version 1.4.4; Rayyan Systems Inc., Cambridge, MA) was utilized. This tool helped in examining the titles and abstracts of the studies to determine their relevance to the review question. Studies deemed pertinent were then selected for full-text retrieval. The inclusion of studies was evaluated based on predefined criteria to ensure that only those meeting the review's focus and quality standards were included. This structured approach ensured a thorough and methodical selection of relevant literature for the review. Reasons for excluding publications read in the full text were described in the appendix (Table 6) in the review. The number of records from the searches and the selection process was depicted in a Preferred Reporting Items for Systematic Reviews and Meta-Analyses (PRISMA) flow diagram (Figure 1). In the review process, screening, selection, and evaluation of studies were conducted by two independent reviewers. The initial screening involved assessing titles and abstracts to determine relevance based on predefined criteria. Both reviewers then independently evaluated the full-text articles for final inclusion. Additionally, both reviewers assessed the methodological quality of the included studies. This approach aimed to ensure thoroughness and reduce potential bias, aligning with best practices for systematic reviews.

Data Extraction

The data extraction process is essential for ensuring comprehensive analysis. The process starts with creating a standardized extraction form to capture key details from each study, including authors, publication year, and source. Essential data include the study design, setting, and duration, with a specific focus on rural and underserved areas. Detailed information about the population, such as sample size and demographics, as well as the intervention's description, technology used, and duration, is collected. Comparisons and outcomes are crucial, documenting the control group and any primary and secondary outcomes, along with the measurement tools and key findings. Challenges and barriers related to telehealth in these areas are noted, alongside the study’s methodological quality [20,21].

Quality Assessment

The Cochrane Risk-of-Bias tool for randomized trials (RoB 2) is designed to assess the RoB in randomized controlled trials (RCTs), focusing on five key domains that could affect the validity and reliability of study results. The first domain evaluates the randomization process, examining how participants were assigned to different intervention groups to ensure comparability and minimize selection bias. The second domain deviations from intended intervention look at whether participants adhered to their assigned interventions and if deviations could influence the outcomes. The third domain addresses missing outcome data, assessing how the study handled any missing data and whether the methods used to address it could introduce bias. The fourth domain, outcome measurement, assesses the accuracy and consistency of outcome measurement, including whether outcome assessors were blinded and if measurement methods were reliable. Lastly, the selection of the reported result domain examines whether the reported results reflect all pre-specified outcomes and analyses, scrutinizing for any selective reporting biases. Each domain is judged as having a low RoB, some concerns, or a high RoB based on specific signaling questions. This comprehensive assessment helps determine the overall RoB in a study, guiding the interpretation of its findings and assessing its reliability [22-24].

Analysis and Synthesis of Data

Systematically organizing and presenting the results from the studies included describing how telehealth interventions affected key outcomes such as quality of life, symptom management, and patient satisfaction. It also involved highlighting the various challenges and barriers encountered in implementing telehealth solutions in rural and underserved regions, such as issues with technology access, training, and logistical constraints. The review was able to provide a rich, contextualized summary of the evidence, offering insights into both the benefits and limitations of telehealth in palliative care settings. This approach helps identify common themes and issues across studies, providing a clearer picture of how telehealth performs in these specific contexts and guiding future research and practice improvements.

Review findings

Table 4 summarizes the characteristics and key findings of the studies included in the systematic review, providing an overview of the study designs, populations, interventions, outcomes, and major conclusions drawn from each study.

**Table 4:** Characteristics and findings of studies included in the systematic review

Author/Year	Objectives	Outcome	Population	Condition	Findings	Challenges
Head et al. 2017 [25]	To review patient-reported outcomes of telehealth in palliative care	Patient-reported outcomes	Mixed	Palliative care	Telehealth interventions have been found to improve symptom management and enhance overall quality of life for patients by providing timely and effective remote support and monitoring.	Differences in the types of telehealth interventions and how outcomes are measured make it difficult to compare results.
Jiang et al. 2023 [26]	To evaluate the feasibility of telehealth-assisted home-based specialist palliative care in rural Australia	Feasibility and acceptability	Rural	Palliative care	Telehealth has proven to be feasible and well-accepted in rural areas, improving access to care for individuals who otherwise might have limited healthcare options.	Small sample sizes and selection bias can affect the reliability and generalizability of study findings.
Bonsignore et al., 2018 [27]	To assess the feasibility and acceptability of a telehealth program in rural palliative care	Feasibility and acceptability	Rural	Palliative care	Telehealth interventions are generally well-received and practical, and they significantly enhance access to care for patients in underserved or remote locations.	Studies with small sample sizes and brief durations may not provide robust or long-term insights.
Steindal et al., 2023 [28]	To review advantages and challenges of telehealth for home-based palliative care	Advantages and challenges	Mixed	Palliative care	Despite its benefits, telehealth faces challenges related to integrating with existing care systems and technological issues, which can affect its overall effectiveness.	Differences in study designs and implementation approaches can affect the consistency of findings.
Zheng et al., 2016 [29]	To review caregiver outcomes related to telehealth in palliative care.	Caregiver outcomes	Mixed	Palliative care	Telehealth interventions have positively affected caregiver burden by providing better support and reducing stress through remote consultations and guidance.	Differences in telehealth interventions and how outcomes are measured create variability in results.
Allen Watts et al., 2021 [30]	To improve palliative care access through telehealth	Access and Effectiveness	Mixed	Palliative care	Telehealth has enhanced patient access to care and improved communication between patients and healthcare providers, facilitating better management of health conditions.	Findings may not be generalizable due to limited sample diversity or inherent biases in sample selection.
Sebastian et al., 2024 [31]	Evaluate the effectiveness of integrated palliative care telehealth intervention in chronic heart failure	Effectiveness of telehealth intervention	Mixed	Chronic heart failure	Telehealth interventions have been shown to improve palliative care outcomes, such as symptom management and patient comfort.	Different types of interventions and outcomes lead to inconsistencies across studies.
Biemba et al., 2020 [32]	Assess the impact of mobile health-enhanced supervision and supply chain on community case management of common illnesses	Improvement in case management for malaria, diarrhoea, and pneumonia	Rural	Malaria, diarrhea, pneumonia	Improved supervision and management of supply chains have led to better-integrated community case management of common illnesses.	Implementing telehealth in rural areas may face specific challenges such as limited resources or infrastructure.
Alacevich et al., 2024 [33]	Connect patients with cancer-induced financial toxicity to telehealth financial counseling	Impact on managing financial toxicity	Mixed	Cancer-induced financial toxicity	Telehealth-based financial counseling has improved the management of financial challenges associated with cancer treatment.	Small sample sizes and short study periods can limit the validity and applicability of the results.
Rimmer et al., 2018 [34]	Compare clinic-based vs. home-based telerehabilitation for MS patients	Effectiveness of clinic vs. home-based interventions	Rural	Multiple sclerosis	Home-based telehealth interventions provide greater flexibility and can be as effective as clinic-based options.	Differences in patient adherence and engagement can influence the effectiveness of telehealth interventions.
Kapinos 2019 [35]	Explore use and experiences with telelactation among rural breastfeeding mothers	Effectiveness and user experience with telelactation	Rural	Breastfeeding support	Telelactation services are well received and effective in assisting breastfeeding in rural areas.	Technology and internet access issues can hinder the effectiveness and reach of telehealth interventions.
Parker et al. 2022 [36]	Prevent chronic disease in overweight/obese patients with low health literacy using eHealth	Impact on chronic disease prevention	Urban	Overweight, obesity	E-health interventions have been successful in preventing chronic diseases among patients with low health literacy.	Challenges related to technology use and varying levels of health literacy can affect the success of telehealth programs.
Namasivayam et al., 2022 [37]	Review the use of telehealth for after-hours palliative care services in rural Australia	Effectiveness of telehealth in providing after-hours palliative care	Rural	Palliative care	Telehealth is effective in providing after-hours palliative care, particularly beneficial for patients in rural or remote areas who need access to care outside of regular office hours.	Limited access to technology and related challenges can impact the effectiveness of telehealth services.
Gordon et al., 2022 [38]	Review leveraging telehealth for palliative care delivery to remote communities	Effectiveness of telehealth for palliative care in remote communities	Rural	Palliative care	Telehealth significantly improves access to palliative care services in remote areas, ensuring that patients receive necessary care despite geographic barriers.	Remote settings may face unique barriers that affect the implementation of telehealth interventions.
Imam et al. 2024 [39]	Examine the evolution of telehealth and its impact on palliative care and medication management	Impact of telehealth on palliative care and medication management	Mixed	Palliative care, medication	Telehealth has a considerable impact on enhancing palliative care and medication management by improving both accessibility and continuity of care, leading to better patient outcomes.	Differences in how telehealth is implemented can lead to variable outcomes and effectiveness.
Bekelman et al., 2024 [40]	Evaluate the impact of a palliative telecare team on the quality of life in patients with COPD, heart failure, or interstitial lung disease	Quality of life	Mixed	COPD, Hheart failure, interstitial lung disease	Telecare interventions have been associated with improved quality of life, providing patients with better support and management of their health conditions through remote care solutions.	Challenges in implementing telehealth and ensuring adherence can impact the overall success of the intervention.
Dionne-Odom et al., 2022 [41]	Assess the effectiveness of a lay navigator-led early palliative care intervention for African American and rural caregivers of individuals with advanced cancer	Caregiver well-being	Rural	Advanced cancer	Telehealth has positively affected caregiver well-being by providing better support, reducing caregiver stress, and improving overall caregiving experiences.	Difficulties in recruiting participants and cultural differences can impact the study's outcomes and applicability.
Ha et al., 2023 [42]	Examine effects of telemedicine-based inspiratory muscle training and walking promotion in lung cancer survivors	Physical function, walking ability	Mixed	Lung cancer	The smartphone app served as a tool to enhance the cardiac rehabilitation process by providing structured support and feedback. This likely led to improved physical function and walking ability among patients.	Studies with small sample sizes and short durations may not provide comprehensive data.
Lozano-Lozano et al., 2023 [43]	Assess the impact of mHealth plus occupational therapy on cognitive function, mood, and physical function in cancer survivors	Cognitive function, mood, physical function	Mixed	Post-cancer recovery	mHealth interventions combined with occupational therapy have improved cognitive and physical functions in cancer patients.	Difficulty in integrating data from various sources can hinder the analysis and application of findings.
Nipp et al., 2022 [44]	Investigate the effects of perioperative geriatric intervention for older adults with cancer	Geriatric health outcomes	Mixed	Cancer in older adults	Perioperative geriatric interventions have led to improved health outcomes in older adults with cancer.	Differences in how interventions are applied can lead to inconsistent results.
Okunade et al., 2024 [45]	Study impact of mobile health technologies on HPV vaccination uptake among mothers of unvaccinated girls	HPV vaccination uptake	Urban	HPV vaccination	Mobile health technologies are expected to improve vaccination rates among young girls, highlighting the potential of digital tools in promoting public health.	Challenges in implementing interventions and conducting follow-ups can affect study outcomes.
Schuit et al., 2022 [46]	Evaluate the cost-utility of eHealth application 'Oncokompas' for cancer patients' self-management	Cancer-related symptom management	Mixed	Incurably ill cancer patients	Telehealth interventions have been effective in helping patients manage their symptoms, providing them with tools and support for better self-care	High costs and issues with technology adoption can limit the implementation and effectiveness of telehealth services.
Rainsford et al., 2022 [47]	Examine the effectiveness of telehealth palliative care Needs Rounds in rural aged care during COVID-19	Palliative care needs	Rural	Palliative care	Telehealth has proven to be an effective method for delivering palliative care services, improving overall care and patient satisfaction.	Issues with technology access and specific challenges in rural areas can impact the success of telehealth programs.
Bakitas et al., 2020 [48]	Evaluate early palliative care telehealth vs usual care for heart failure	Improved quality of life and symptom management in the intervention group	Urban	Heart failure	Telehealth interventions have resulted in improved patient outcomes compared to traditional care methods, demonstrating their efficacy in enhancing healthcare delivery.	Differences in patient adherence and access to technology can influence the effectiveness of telehealth services.
Casillas et al., 2019 [49]	Assess mobile technology and peer navigation for AYA cancer survivorship	Enhanced survivorship care engagement and support for AYA patients	Mixed	Cancer	Mobile technology and peer navigation have improved care for adolescent and young adult cancer survivors, providing valuable support and resources through digital means.	Limited generalizability and variability in technology acceptance can affect the applicability of findings.
Dionne-Odom et al., 2020 [50]	Examine telehealth palliative care for family caregivers of advanced heart failure	Improved caregiver support and reduced stress	Urban	Advanced heart failure	Telehealth interventions have improved support for caregivers, helping to reduce their stress and improve their caregiving experiences.	Issues with caregiver engagement and technology barriers can impact the success of telehealth interventions.
Kamal et al., 2019 [51]	Assess the usability of PCforMe in advanced cancer patients	Enhanced usability and patient engagement with the intervention	Urban	Advanced cancer	The PC fo rMe intervention has improved usability and engagement among advanced cancer patients, offering a more user-friendly and interactive experience.	A limited patient population and usability issues can affect the overall findings of the study.
Lunde et al., 2020 [52]	Evaluate long-term follow-up with a smartphone app post-cardiac rehabilitation	Improved exercise capacity and adherence to rehabilitation	Mixed	Post-cardiac rehabilitation	The use of a smartphone app has led to better exercise capacity following cardiac rehabilitation, demonstrating the benefits of mobile technology in supporting physical recovery.	Variability in technology acceptance and follow-up adherence can affect the effectiveness of telehealth services.
Ngoma et al., 2021 [53]	Examine a mobile solution for palliative care coordination in Tanzania	Improved coordination and patient management in palliative care	Rural	Palliative care	A mobile solution has improved palliative care coordination for patients in Tanzania, enhancing the management of care through digital tools.	Inadequate technology infrastructure and cultural barriers can affect the implementation and success of telehealth.
Vaughan et al., 2021 [54]	Investigate telehealth-supported integrated care for diabetes	Improved HbA1c levels with telehealth support and medication access	Mixed	Diabetes	Telehealth-supported integrated care has improved diabetes management, showing the effectiveness of remote care in managing chronic conditions.	Challenges in integrating community health workers (CHWs) and variability in medication access can affect outcomes.

Table 5 presents the Cochrane RoB assessment for studies included in the systematic review, evaluating potential biases across key domains such as selection, performance, detection, and reporting. This assessment provides a comprehensive overview of the methodological quality of the studies, highlighting areas of low, high, or unclear RoB. The results inform the overall confidence in the review's findings and the reliability of the evidence presented.

**Table 5:** Cochrane risk of bias assessment of studies included in the systematic review

Study	Randomization Process	Deviations from Intended Intervention	Missing Outcome Data	Outcome Measurement	Selection of Reported Result	Overall Quality (RoB)
Head et al. 2017 [25]	Low	Some concerns	Low	Low	Low	Low
Jiang et al. 2023 [26]	Low	Low	Some concerns	Low	Low	Low
Bonsignore et al. 2018 [27]	Low	Some concerns	Low	Some concerns	Low	Some concerns
Steindal et al. 2023 [28]	Low	Low	Low	Low	Some concerns	Low
Zheng et al. 2016 [29]	Low	Low	Some concerns	Low	Low	Low
Allen Watts et al. 2021 [30]	Low	Some concerns	Low	Some concerns	Low	Some concerns
Sebastian et al. 2024 [31]	Some concerns	Some concerns	Low	Some concerns	Some concerns	Some concerns
Biemba et al. 2020 [32]	Low	Low	Some concerns	Low	Low	Low
Alacevich et al. 2024 [33]	Low	Low	Low	Low	Low	Low
Rimmer et al. 2018 [34]	Low	Some concerns	Some concerns	Low	Some concerns	Some concerns
Kapinos 2019 [35]	Low	Low	Low	Low	Low	Low
Parker et al. 2022 [36]	Low	Some concerns	Low	Low	Some concerns	Some concerns
Namasivayam et al. 2022 [37]	Low	Low	Some concerns	Low	Low	Low
Gordon et al. 2022 [38]	Low	Low	Some concerns	Low	Low	Low
Imam et al. 2024 [39]	Low	Low	Low	Low	Low	Low
Bekelman et al. 2024 [40]	Low	Some concerns	Low	Low	Some concerns	Some concerns
Dionne-Odom et al. 2022 [41]	Low	Low	Low	Low	Some concerns	Low
Ha et al. 2023 [42]	Low	Low	Some concerns	Low	Low	Low
Lozano et al. 2023 [43]	Low	Low	Low	Some concerns	Low	Low
Nipp et al. 2022 [44]	Low	Low	Some concerns	Low	Low	Low
Okunade et al. 2024 [45]	Low	Low	Some concerns	Low	Low	Low
Schuit et al. 2022 [46]	Low	Low	Low	Some concerns	Low	Low
Rainsford et al. 2022 [47]	Low	Some concerns	Low	Low	Low	Some concerns
Bakitas et al. 2020 [48]	Low	Some concerns	Some concerns	Low	Low	Some concerns
Casillas et al. 2019 [49]	Low	Low	Low	Low	Low	Low
Dionne-Odom et al. 2020 [50]	Low	Low	Low	Low	Low	Low
Kamal et al. 2019 [51]	Low	Low	Some concerns	Low	Low	Low
Lunde et al. 2020 [52]	Low	Some concerns	Low	Some concerns	Low	Some concerns
Ngoma et al. 2021 [53]	Low	Low	Some concerns	Low	Low	Low
Vaughan et al. 2021 [54]	Low	Some concerns	Low	Low	Some concerns	Some concerns

The overall RoB rating is derived from the assessments across the five domains. A study rated as "low" across all domains generally receives an overall "low risk" rating, indicating robust methodological quality. Conversely, studies with "some concerns" or "high risk" in one or more domains may have an overall rating of "some concerns" or "high risk," reflecting potential biases or limitations.

Table 5 examines the RoB in studies including systematic reviews. In assessing the RoB for the evaluations, five domains are summarized as follows: the study by Head et al. was rated as having a low RoB overall, with low risk in the randomization process, outcome measurement, and selection of reported results, though it had some concerns regarding deviations from the intended intervention and missing outcome data [25]. The study by Jiang et al. also received a low overall RoB, with low ratings in the randomization process, outcome measurement, and selection of reported results, but some concerns regarding missing outcome data [26]. The study by Bonsignore et al. presented a mixed profile, with low risk in randomization and missing outcome data, but some concerns about deviations from the intended intervention, outcome measurement, and selection of reported results [27]. The study by Steindal et al. was rated as having a low overall RoB, with low ratings across most domains, except for some concerns in the selection of reported results [28]. The study by Zheng et al. showed a low RoB overall, with low ratings in randomization, deviations from intended intervention, and selection of reported results, but some concerns regarding missing outcome data [29]. The study by Allen Watts et al. had a low overall RoB with low scores in randomization, missing outcome data, and selection of reported results, though some concerns were noted in deviations from the intended intervention and outcome measurement [30]. The study by Sebastian et al. identified some concerns overall, showing mixed ratings with some concerns in most domains [31]. The study by Biemba et al. had a low RoB overall, with low ratings across most domains [32]. The study by Alacevich et al. rated as having a low RoB in all domains [33]. The study by Rimmer et al. had an overall risk of some concerns, with low ratings in several domains, but some concerns in deviations from the intended intervention and selection of reported results [34]. The study by Kapinos et al. was rated with a low RoB overall, with low scores across all domains [35]. The study by Parker et al. had some concerns overall, with low ratings in several domains but some concerns in deviations from the intended intervention and selection of reported results [36]. The study by Namasivayam et al. was rated as having a low RoB overall, with low ratings in most domains but some concerns about missing outcome data [37]. Gordon et al. also received a low RoB overall, with low ratings across most domains, except for some concerns about missing outcome data [38]. The study by Imam et al. was rated as having a low RoB in all domains [39]. The study by Bekelman et al. showed some concerns overall, with mixed ratings across the domains [40]. The study by Dionne-Odom et al. had a low RoB overall with low ratings across all domains, except for some concerns in the selection of reported results [41]. The study by Ha et al. was rated as having a low RoB overall, with low ratings in most domains but some concerns about missing outcome data [42]. The study by Lozano et al. had a low overall RoB, with low ratings in most domains, except for some concerns in outcome measurement [43]. The study by Nipp et al. received a low RoB overall, with low ratings in most domains but some concerns about missing outcome data [44]. The study by Okunade et al. had a low RoB overall, with low ratings across all domains, except for some concerns about missing outcome data [45]. The study by Schuit et al. was rated with a low RoB overall, except for some concerns in outcome measurement [46]. The study by Rainsford et al. had some concerns overall, with low ratings in most domains but some concerns in deviations from the intended intervention [47]. The study by Bakitas et al. 2020 showed some concerns overall, with mixed ratings across domains [48]. The studies by Casillas et al. and Dionne-Odom et al. were both rated with a low RoB overall, with low ratings across all domains [49,50]. The study by Kamal et al. also had a low RoB overall, with low ratings, except for some concerns about missing outcome data [51]. Lunde et al. had some concerns overall, with low ratings in most domains, except for deviations from the intended intervention [52]. The study by Ngoma et al. was rated as having a low RoB overall, with low ratings in most domains but some concerns about missing outcome data [53]. The study by Vaughan et al. had an overall rating of some concerns, with low ratings in most domains but some concerns in deviations from the intended intervention and selection of reported results [54]. The overall RoB rating is derived from these domain-specific assessments. Studies rated as "Low" across all domains generally indicate robust methodological quality, while those with "Some Concerns" or "High Risk" in one or more domains reflect potential biases or limitations.

The systematic review provides a comprehensive analysis of the effectiveness and challenges associated with telehealth in palliative care, revealing both its potential benefits and significant limitations. Telehealth consistently demonstrated improvements in symptom management and quality of life, particularly in home-based settings where it was found to be feasible and acceptable. It also facilitated better access to care, especially in remote and underserved communities, and showed a positive impact on reducing caregiver burden. However, the review identified several challenges, including small sample sizes, selection bias, and significant technological and infrastructure barriers, particularly in rural or resource-limited settings. There was also considerable variability in interventions and outcomes, complicating the ability to draw definitive conclusions about the overall effectiveness of telehealth in palliative care. Cost and adoption issues, particularly in applications such as the 'Oncokompas' for symptom management, further limited broader implementation. Cultural and recruitment challenges, especially in low-resource settings, also posed significant hurdles. The effectiveness of telehealth varied depending on the specific application and context, with some interventions, such as perioperative care, showing improved outcomes but with inconsistent results. Additionally, patient and caregiver engagement varied, with some studies reporting high levels of engagement, while others faced significant challenges. Despite these issues, the review underscores the potential of telehealth to enhance palliative care, though it emphasizes the need to address the identified challenges to fully optimize its impact. The exclusion of non-relevant studies ensured a focused review, but the diversity of the included studies still presents a challenge in synthesizing the findings into a cohesive narrative.

Effectiveness of Telehealth in Rural and Underserved Areas

Improved access to care: Telehealth has demonstrated a substantial impact on improving access to palliative care for patients in rural and underserved areas. Studies such as those of Jiang et al. and Bonsignore et al. provide compelling evidence of this benefit [26,27]. Jiang et al. focused on the feasibility and acceptability of telehealth-assisted palliative care in rural Australia, showing that telehealth services bridge gaps between patients and specialized care providers. Similarly, Bonsignore et al. assessed the feasibility and acceptability of telehealth programs in rural palliative care, finding that such interventions effectively enhance service availability in regions with limited healthcare infrastructure [27]. The effectiveness of telehealth in improving access is critical because rural and underserved areas often face challenges such as a shortage of healthcare providers and limited healthcare facilities. By enabling remote consultations and follow-ups, telehealth mitigates geographical barriers and ensures that patients receive necessary palliative care despite physical distance. This enhanced access is crucial in addressing disparities in healthcare availability and ensuring that patients in these areas have equitable access to specialized care.

Enhanced symptom management: Telehealth interventions have been shown to facilitate better symptom management through timely remote consultations and continuous monitoring. According to Head et al. and Steindal et al., telehealth allows for frequent adjustments to care plans based on real-time data, leading to improved symptom management and overall patient comfort [25,28]. Head et al. highlighted that patient-reported outcomes improved with telehealth support, reflecting better symptom control and management [25]. Steindal et al. reviewed the advantages and challenges of telehealth, noting that remote support enables prompt modifications to care strategies, which is particularly beneficial for managing complex palliative care needs [28]. Enhanced symptom management is a critical outcome of telehealth, as it directly impacts patient comfort and quality of life. In palliative care, where managing symptoms is a primary goal, the ability to make timely adjustments to treatment plans remotely is invaluable. Telehealth facilitates ongoing communication between patients and providers, ensuring that symptoms are managed more effectively and that patients receive the support they need immediately.

Increased patient and caregiver satisfaction: Telehealth has been associated with increased satisfaction among both patients and caregivers. Zheng et al. and Allen Watts et al. found that telehealth enhances communication between patients and healthcare providers, contributing to a more positive experience and greater satisfaction with care [29,30]. Watts et al. highlighted that telehealth improved patient access to care and facilitated better communication, leading to higher satisfaction levels [29]. Zheng et al. reported that telehealth interventions positively affected caregiver outcomes by reducing stress and providing better support [30]. Patient and caregiver satisfaction is a key indicator of the success of any healthcare intervention. Telehealth’s ability to improve communication and provide support remotely contributes to a more favorable experience for both patients and caregivers. High satisfaction levels are indicative of effective care delivery and can lead to better adherence to treatment plans and improved overall outcomes. The convenience and responsiveness of telehealth services address the emotional and practical needs of patients and caregivers, thereby enhancing their overall experience.

Cost-effectiveness: Telehealth's cost-effectiveness in delivering palliative care, particularly in rural and underserved areas, is a notable benefit. The studies by Biemba et al. and Alacevich et al. highlight how telehealth can reduce costs by minimizing the need for travel and in-person visits [32,33]. Biemba et al. explored the impact of mobile health-enhanced supervision on community case management for common illnesses and found that telehealth solutions can streamline care and reduce operational costs [32]. Alacevich et al. focused on telehealth-based financial counselling for cancer patients and demonstrated that remote consultations could alleviate some of the financial pressures associated with cancer treatment, contributing to overall cost savings [33]. Telehealth offers a cost-effective solution for palliative care, particularly in rural and underserved areas. Traditional models often involve significant travel expenses and strain resources due to frequent in-person visits. Telehealth eliminates this burden, allowing providers to manage multiple patients more efficiently. This efficiency not only benefits patients by lowering out-of-pocket expenses but also optimizes resource allocation and reduces overall care delivery costs. Telehealth is a viable option for expanding access to palliative care in areas with financial and logistical barriers.

Challenges of Telehealth in Rural and Underserved Areas

Differences in intervention types and measurement: Different types of interventions and outcomes lead to inconsistencies across studies. The diversity in telehealth interventions - ranging from video consultations to remote monitoring, coupled with varying methods for outcome measurement - makes it difficult to draw consistent conclusions across studies. For instance, Head et al. highlighted that different telehealth tools and measurement approaches yield inconsistent results, complicating efforts to compare the effectiveness of these interventions [21]. Similarly, Bonsignore et al. pointed out that varied approaches to telehealth implementation could lead to disparate outcomes, creating a challenge for synthesizing results and formulating generalizable recommendations [27].

Sample Size and Study Duration

Small sample sizes and short study durations limit the reliability and applicability of findings. Jiang et al. noted that small sample sizes could skew results and affect the generalizability of findings, as they may not represent broader population trends [26]. Steindal et al. also pointed out that short study durations might not capture the long-term effects of telehealth interventions, undermining the ability to assess their sustainability and long-term benefits [28]. This limitation is evident in studies such as those by Allen Watts et al., where brief study periods may not reflect enduring impacts [30].

Study Design and Implementation

Variability in study design and implementation approaches contributes to inconsistencies in findings. Zheng et al. and Sebastian et al. emphasized that differences in how telehealth interventions are designed and applied can lead to varied outcomes [29,31]. This variability affects the ability to compare studies and draw generalized conclusions. For example, different telehealth models - such as synchronous versus asynchronous consultations - may yield different results, influencing the overall assessment of telehealth efficacy.

Technology and Infrastructure

Inadequate technology and infrastructure, particularly in rural areas, pose significant barriers to effective telehealth implementation. Lozano et al. and Ngoma et al. noted that limited access to reliable technology and internet services can hinder the effectiveness of telehealth interventions [43,53]. This challenge is compounded in rural settings where infrastructure may be lacking, making it difficult to deliver consistent and high-quality care. Technology access issues can also exacerbate existing disparities in healthcare access and quality.

Patient Engagement and Adherence

Variability in patient engagement and adherence can significantly impact the success of telehealth interventions. Ha et al. and Bakitas et al. found that inconsistent patient adherence to telehealth protocols affects the overall effectiveness of interventions [42,48]. Factors such as technological literacy, personal motivation, and perceived value of telehealth services play a role in determining patient engagement. Low engagement can undermine the potential benefits of telehealth, affecting outcomes such as symptom management and quality of life.

Cultural and Contextual Factors

Cultural differences and varying levels of technology use can affect the success of telehealth interventions. Alacevich et al. highlighted those cultural barriers, such as stigma or lack of familiarity with technology, can impede the effectiveness of telehealth services [33]. Namasivayam et al. and Kamal et al. found that health literacy and cultural attitudes towards telehealth influence its adoption and efficacy [37,51]. Addressing these factors is crucial for ensuring that telehealth services are culturally sensitive and accessible to diverse populations.

Generalizability and Bias

The generalizability of findings is often limited by inherent biases in sample selection. Bekelman et al. and Vaughan et al. discussed how specific characteristics of study populations - such as age, socioeconomic status, and geographic location - can affect the applicability of results to broader populations [40,54]. Limited sample diversity can lead to skewed findings that do not reflect the experiences of different demographic groups, thus affecting the overall utility of telehealth interventions.

Cost and Adoption

High costs and challenges in technology adoption can restrict the implementation and effectiveness of telehealth services. Schuit et al. and Casillas et al. pointed out that the financial burden of telehealth technology and issues with widespread adoption can limit the reach and impact of these interventions [46,49]. High costs can be a barrier to both providers and patients, while difficulties in integrating telehealth solutions into existing systems can affect the overall efficiency and effectiveness of care.

Quality of Telehealth Infrastructure

The quality and reliability of telehealth infrastructure, including software and hardware, can significantly impact the effectiveness of telehealth interventions. Okunade et al. and Schuit et al. emphasized that technical issues, such as software glitches, poor video quality, and hardware malfunctions, can undermine the delivery of care and patient satisfaction [45,46]. Ensuring robust and reliable telehealth infrastructure is essential for providing effective remote care and maintaining consistent service quality.

Patient and Caregiver Acceptance

Acceptance and satisfaction of telehealth services by patients and caregivers can influence the overall success of these interventions. Dionne-Odom et al. and Kamal et al. noted that resistance to adopting telehealth solutions, whether due to technological discomfort or preference for in-person interactions, can affect the effectiveness of remote care [50,51]. Ensuring that patients and caregivers are comfortable with and receptive to telehealth is crucial for maximizing its benefits.

Training and Support for Healthcare Providers

The effectiveness of telehealth interventions is often dependent on the level of training and support provided to healthcare professionals. Steindal et al. and Bekelman et al. found that inadequate training for providers can lead to suboptimal use of telehealth technologies and affect the quality of care [28,40]. Effective telehealth implementation requires comprehensive training programs to ensure that healthcare providers are skilled in using telehealth platforms and delivering high-quality remote care. Each of these challenges highlights critical areas where telehealth interventions in palliative care need improvement. Addressing these issues is essential for optimizing telehealth services, ensuring equitable access, and enhancing patient outcomes.

Discussion

Telehealth in rural and underserved areas holds significant promise for enhancing access to palliative care, improving quality of life, and managing symptoms. However, its effectiveness is often constrained by challenges such as limited internet access, low technological literacy, and inadequate infrastructure [55]. This review underscores the necessity for targeted strategies to address these barriers and integrate telehealth effectively into existing palliative care models. By examining various challenges, including technology access, training issues, and logistical constraints, the review provides a contextualized summary of the evidence. It highlights both the benefits, such as improved care delivery and patient outcomes, and the limitations, including methodological weaknesses and practical difficulties. The review identifies common themes and issues across studies, offering a clearer understanding of telehealth’s performance in these settings. Practical implications are discussed, focusing on real-world applications and addressing specific challenges encountered. Additionally, research gaps are highlighted, pointing to the need for larger trials, further exploration of telehealth aspects, and long-term outcome studies. These insights guide future research and practice, aiming to advance the integration and effectiveness of telehealth in palliative care.

Burner-Fritsch et al. focused on the usability and integration of electronic patient-reported outcome measurements (EPROMs) in palliative home care, emphasizing the need for user-centered design and addressing varying levels of digital literacy [56]. Their findings reveal challenges related to technology adoption among patients and caregivers, highlighting the importance of feedback in the design process. Gordon et al. explored telehealth’s role in improving access to palliative care in remote communities [38]. Burner-Fritsch et al. [56] and Gordon et al. [38] both examined the challenges of implementing telehealth, though from distinct angles. Burner-Fritsch et al. focused on the usability and integration of EPROMs in palliative home care, highlighting the importance of user-centered design and digital literacy. They identify specific challenges related to technology adoption among patients and caregivers, emphasizing the need for feedback in the design process. In contrast, Gordon et al. explored telehealth's broader role in improving access to palliative care in remote communities, finding that, while telehealth enhances care access and symptom management, it also faces significant technological barriers and variability in implementation. While both studies recognize the importance of technology integration, Burner-Fritsch et al. offered a more focused examination of design and usability issues, whereas Gordon et al. addressed logistical and systemic challenges on a broader scale.

DeMayo et al. studied the impact of telehealth on pediatric healthcare providers’ well-being, finding that, while telehealth improves work-life balance, it introduces new stressors such as maintaining professional boundaries and managing technology issues [57]. Dionne-Odom et al. examined factors affecting the implementation of critical care telemedicine, identifying technology barriers, integration challenges, and stakeholder engagement issues [41]. DeMayo et al. and Dionne-Odom et al. explored the effects of telehealth on healthcare providers, but their focus areas differ. DeMayo et al. study the impact on pediatric healthcare providers’ well-being, finding that telehealth improves work-life balance but introduces new stressors, such as maintaining professional boundaries and managing technology. Dionne-Odom et al., on the other hand, examined factors affecting the implementation of critical care telemedicine, identifying technology barriers, integration challenges, and issues with stakeholder engagement. While DeMayo et al. focused specifically on the well-being of pediatric providers, emphasizing work-life balance and stressors unique to pediatric care, Dionne-Odom et al. took a broader approach, addressing a wide range of implementation challenges and the perceptions of stakeholders in critical care settings.

Gatter et al. provided a protocol for evaluating telemedicine in neurological consultations within palliative care, focusing on the need for rigorous trials to assess effectiveness [58]. Johnson et al. investigated the use of mobile health apps and wearables to monitor pain in sickle cell disease, highlighting their potential and challenges in data integration and user engagement [13]. Gatter et al. [58] and Johnson et al. [13] both evaluated telehealth tools but with different focuses. Gatter et al. provided a protocol for evaluating telemedicine in neurological consultations within palliative care, emphasizing the need for rigorous trials to assess effectiveness. Their work is forward-looking, aiming to establish a standardized approach for evaluating telemedicine in a specialized area. In contrast, Johnson et al. [13] investigated the use of mobile health apps and wearables for monitoring pain in sickle cell disease, highlighting both the potential of these technologies and the challenges related to data integration and user engagement. While Gatter et al. [58] are concerned with the design and execution of a trial to assess telemedicine’s effectiveness in a specific context, Johnson et al. [13] deal with the practical challenges of implementing and engaging users with existing mobile technologies.

Lomenick et al. analyzed the economic benefits of telemedicine in hospice care for rural areas, emphasizing cost reduction and improved accessibility [59]. Bekelman et al. [40] explored how telemedically augmented palliative care empowers patients with advanced cancer and their caregivers, highlighting improvements in access to information and support [40]. Lomenick et al. [59] and Bekelman et al. [40] explored the benefits of telehealth in palliative care from different perspectives. Lomenick et al. [59] focused on the economic benefits of telemedicine in hospice care for rural areas, emphasizing cost reduction and improved accessibility. Their analysis is centered on the financial impact and cost-effectiveness of telehealth interventions. In contrast, Bekelman et al. [59] discussed how telemedically augmented palliative care empowers patients with advanced cancer and their caregivers, highlighting improvements in access to information and support. While both studies demonstrate the benefits of telehealth, Lomenick et al. [59] emphasized economic implications, whereas Bekelman et al. [40] focused on qualitative aspects of patient and caregiver experiences, particularly in terms of empowerment and support.

Prell et al. provided recommendations for network care standards for Parkinson's disease, including telemedicine, emphasizing the need for standardized protocols [60]. Imam et al. reviewed the role of telehealth in palliative care during the COVID-19 pandemic, highlighting its role in maintaining care continuity and addressing technology access issues [39]. Prell et al. [60] and Imam et al. [39] contributed to understanding telemedicine in palliative care but focused on different aspects. Prell et al. [60] provided recommendations for network care standards for Parkinson's disease, including telemedicine, emphasizing the need for standardized protocols. Their study is disease-specific and aims to establish clear guidelines for telehealth implementation in Parkinson’s care. On the other hand, Imam et al. [39] reviewed the role of telehealth in palliative care during the COVID-19 pandemic, highlighting its role in maintaining care continuity and addressing technology access issues. Their review provides a broader perspective, focusing on the challenges and opportunities presented by telehealth during a global crisis. While Prell et al. focused on creating standards for telehealth in a specific disease context, Imam et al. [39] provided an overview of telehealth’s broader impact during an unprecedented global event.

Strengths

Telehealth has the potential to improve access to palliative care, particularly in remote or underserved areas, by improving service reach and reducing disparities. It also has economic benefits by lowering costs, especially in rural areas. Telehealth can improve provider well-being by offering flexibility, though it introduces new stressors. Empowering patients and caregivers through better access to information and support is another key benefit.

Limitations and Recommendations

Technological barriers and issues with usability can limit the effectiveness of telehealth interventions. Stressors related to maintaining professional boundaries and managing technology can affect provider well-being. Integration challenges with existing care systems are a common concern. Variability in implementation practices can impact the effectiveness of telehealth. Limited long-term data from feasibility studies calls for more research on sustained outcomes. One notable limitation of this review is the use of fewer than four databases in the systematic search process. While the databases selected were comprehensive, including a broader range of databases could help further reduce selection bias and ensure a more inclusive range of relevant studies. Future reviews should consider incorporating at least four databases to enhance the robustness and generalizability of the findings. To address technological barriers, it is crucial to develop user-friendly tools and support systems, support provider well-being by implementing strategies to manage stressors related to telehealth, establish standardized protocols for telehealth implementation, invest in rigorous trials, enhance user training, and evaluate long-term outcomes to address gaps and improve the integration of telehealth in palliative care.

## Conclusions

Telehealth is a promising advancement in palliative care, offering improved access to specialized services and better symptom management for patients in rural and underserved areas. It enhances communication, reduces the financial burden of travel, and increases patient and caregiver satisfaction. To maximize its potential, telehealth must be carefully implemented with attention to technology, integration, and user-centered design.

## Appendices

**Table 6:** Reasons for the exclusion of studies in the systematic review

Study	Objective	Reason for Exclusion	Justification
Swanson et al. 2016 [61]	Quality assurance in obstetric ultrasound using web-based processes	Irrelevant outcome	The study is centered around quality assurance in obstetric ultrasound, which does not align with the focus on palliative care or telehealth interventions.
Jewer et al. 2019 [62]	Evaluate a mobile telesimulation unit for training practitioners	Irrelevant outcome	The study evaluates a mobile telesimulation unit for training high-acuity procedures, rather than focusing on palliative care or telehealth.
Lind et al. 2003 [63]	Effectiveness of telehealth palliative care needs rounds in rural aged care during COVID-19	Outdated study	Published before last 10 year's cutoff; focused on a specific pandemic context, not broadly applicable to current telehealth palliative care.
Haunet al. 2023 [64]	Evaluate mobile and web-based interventions for managing pain and PTSD symptoms	Irrelevant outcome	The study might evaluate mobile and web-based interventions in general, rather than specifically targeting telehealth interventions tailored to palliative care settings. The effectiveness of telehealth in palliative care often requires a tailored approach to address the unique needs of patients in this context.
Chai et al. 2020 [65]	Investigate a music app to address pain in the emergency department	Irrelevant outcome	The study's scope is too narrow and specific to pain management in an emergency department setting, rather than addressing broader telehealth applications or challenges in palliative care, particularly in rural or underserved areas. Therefore, it does not contribute to understanding the effectiveness and challenges of telehealth in the context of palliative care.
Wardlow et al. 2023 [66]	Telemedicine adoption in high-income countries	Limited to high-income countries	The study is restricted to high-income countries, and not relevant to rural or underserved areas.
Wasp et al. 2023 [67]	Technology integration in urban health systems	Limited to high-income countries	The study focuses on technology integration in urban health systems, not relevant to rural or underserved settings.

**Table 7:** Details of the Cochrane Risk of Bias Tool

	Randomization Process			Deviations from Intended Intervention									Missing Outcome Data				Outcome Measurement				Selection of Reported Result			
Study	1.1	1.2	1.3	2.1.1	2.1.2	2.1.3	2.2.1	2.2.2	2.2.3	2.2.4	2.2.5	2.2.6	3.1	3.2	3.3?	3.4	4.1	4.2	4.3	4.4	4.5	5.1	5.2	5.3
Head et al. 2017 [25]	NI	NI	N	N	N	Y	N	N	Y	PN	PN	Y	Y	PN	Y	Y	N	N	N	Y	Y	N	Y	N
Jiang et al. 2023 [26]	Y	Y	N	N	N	Y	N	NI	Y	PN	PY	Y	Y	NI	Y	Y	N	NI	N	Y	Y	Y	Y	Y
Bonsignore et al. 2018 [27]	Y	Y	N	N	N	Y	N	NI	Y	PY	PY	Y	Y	NI	Y	Y	PY	PY	N	N	N	N	N	N
Steindal et al. 2023 [28]	Y	Y	N	N	N	Y	N	Y	Y	PN	PN	Y	Y	PN	Y	Y	N	N	N	Y	Y	Y	Y	Y
Zheng et al. 2016 [29]	Y	Y	N	N	N	Y	N	NI	Y	PN	PN	Y	Y	PN	Y	Y	N	N	N	Y	Y	Y	Y	Y
Allen Watts et al. 2021 [30]	Y	Y	N	N	N	Y	N	NI	Y	PN	PY	Y	Y	PN	Y	Y	N	N	N	Y	Y	Y	Y	Y
Sebastian et al. 2024 [31]	NI	NI	N	N	NI	Y	N	NI	NI	PN	PY	Y	Y	NI	Y	Y	N	N	N	Y	Y	N	N	N
Biemba et al. 2020 [32]	Y	Y	N	N	N	Y	N	NI	Y	PN	PN	Y	Y	PN	Y	Y	N	N	N	Y	Y	Y	Y	Y
Alacevich et al. 2024 [33]	Y	Y	N	N	N	Y	N	NI	Y	PN	PN	Y	Y	PN	Y	Y	N	N	N	Y	Y	Y	Y	Y
Rimmer et al. 2018 [34]	NI	NI	N	N	NI	Y	N	NI	Y	PN	PN	Y	Y	PN	Y	Y	N	NI	N	Y	Y	Y	N	N
Kapinos et al. 2019 [35]	Y	Y	N	N	N	Y	N	NI	Y	PN	PN	Y	Y	N	Y	Y	N	N	N	Y	Y	Y	Y	Y
Parker et al. 2022 [36]	Y	Y	N	N	N	Y	N	NI	Y	PN	PN	Y	Y	PN	Y	Y	N	NI	N	Y	Y	N	N	N
Namasivayam et al. 2022 [37]	Y	Y	N	N	N	Y	N	NI	Y	PN	PN	Y	Y	PN	Y	Y	N	N	N	Y	Y	Y	Y	Y
Gordon et al. 2022 [38]	Y	Y	N	N	N	Y	N	NI	Y	PN	PN	Y	Y	PN	Y	Y	N	N	N	Y	Y	Y	Y	Y
Imam et al. 2024 [39]	Y	Y	N	N	N	Y	N	NI	Y	PN	PN	Y	Y	PN	Y	Y	N	N	N	Y	Y	Y	Y	Y
Bekelman et al. 2024 [40]	NI	NI	N	N	NI	Y	N	NI	NI	PN	PY	Y	Y	NI	Y	Y	N	N	N	Y	Y	N	N	N
Dionne et al. 2022 [41]	Y	Y	N	N	N	Y	N	NI	Y	PN	PY	Y	Y	PN	Y	Y	N	N	N	Y	Y	Y	Y	Y
Ha et al. 2023 [42]	Y	Y	N	N	N	Y	N	NI	Y	PN	PN	Y	Y	PN	Y	Y	N	N	N	Y	Y	Y	Y	Y
Lozano et al. 2023 [43]	Y	Y	N	N	N	Y	N	NI	Y	PN	PN	Y	Y	PN	Y	Y	N	N	N	Y	Y	Y	Y	Y
Nipp et al. 2022 [44]	Y	Y	N	N	N	Y	N	NI	Y	PN	PN	Y	Y	PN	Y	Y	N	N	N	Y	Y	Y	Y	Y
Okunade et al. 2024 [45]	Y	Y	N	N	N	Y	N	NI	Y	PN	PN	Y	Y	PN	Y	Y	N	N	N	Y	Y	Y	Y	Y
Schuit et al. 2022 [46]	Y	Y	N	N	N	Y	N	NI	Y	PN	PN	Y	Y	PN	Y	Y	N	N	N	Y	Y	Y	Y	Y
Rainsford et al. 2022 [47]	Y	Y	N	N	N	Y	N	NI	Y	PN	PN	Y	Y	PN	Y	Y	N	N	N	Y	Y	Y	Y	Y
Bakitas et al. 2020 [48]	Y	Y	N	N	N	Y	N	NI	Y	PN	PN	Y	Y	PN	Y	Y	N	N	N	Y	Y	Y	Y	Y
Casillas et al. 2019 [49]	Y	Y	N	N	N	Y	N	NI	Y	PN	PN	Y	Y	PN	Y	Y	N	N	N	Y	Y	Y	Y	Y
Dionne-Odom et al. 2020 [50]	Y	Y	N	N	N	Y	N	NI	Y	PN	PN	Y	Y	PN	Y	Y	N	N	N	Y	Y	Y	Y	Y
Kamal et al. 2019 [51]	Y	Y	N	N	N	Y	N	NI	Y	PN	PN	Y	Y	PN	Y	Y	N	N	N	Y	Y	Y	Y	Y
Lunde et al. 2020 [52]	Y	Y	N	N	N	Y	N	NI	Y	PN	PN	Y	Y	PN	Y	Y	N	N	N	Y	Y	Y	Y	Y
Ngoma et al. 2021 [53]	Y	Y	N	N	N	Y	N	NI	Y	PN	PN	Y	Y	PN	Y	Y	N	N	N	Y	Y	Y	Y	Y
Vaughan et al. 2021 [54]	Y	Y	N	N	N	Y	N	NI	Y	PN	PN	Y	Y	PN	Y	Y	N	N	N	Y	Y	Y	Y	Y
